# Real-time classification of causes death using Artificial Intelligence – sensitivity analysis

**DOI:** 10.1093/eurpub/ckac129.574

**Published:** 2022-10-25

**Authors:** P Pita Ferreira, D Godinho Simões, C Pinto de Carvalho, F Duarte, E Fernandes, P Casaca, J Loff, AP Soares, P Pinto Leite, A Peralta-Santos

**Affiliations:** 1 Directorate of Information and Analysis, Directorate-General of Health, Lisbon, Portugal; 2 Public Health Unit of Oeste Norte, Health Administration Region of Lisbon and the Tag, Caldas da Rainha, Portugal; 3 Public Health Unit of Almada-Seixal, Health Administration Region of Lisbon and the Tag, Almada, Portugal; 4 Public Health Unit of Alentejo Litoral, Health Administration Region of Alentejo, Santiago do Cacém, Portugal; 5 Department of Global Health, University of Washington, Seattle, Washington, USA; 6 National School of Public Health, NOVA University of Lisbon, Lisbon, Portugal; 7 On a personal capacity, Portugal

## Abstract

**Background:**

Last year Europe registered >365 000 excess deaths, most from preventable causes. In order to timely track deaths, the Portuguese Directorate-General of Health developed a deep neural network that codifies ICD-10 causes of death (AUTOCOD) by analyzing free text in a death certificate (DC). While the performance of AUTOCOD has been demonstrated, it was not clear if it was sustained during excess mortality periods, when text quality could be lower due to the increased pressure on health services.

**Methods:**

We performed a sensitivity analysis comparing the ICD-10 classifications of 330 098 Portuguese DC by AUTOCOD and by human-coders, from 2016 to 2019. Excess mortality was defined using the EuroMOMO methodology and a sub-analysis in periods of extreme excess (+4 and +6 SD). We compared the periods without excess mortality with the periods of excess and extreme mortality by chapter. The same analysis was performed for ICD-10 blocks, for the three most common chapters (neoplasms; diseases circulatory and respiratory system). The confusion matrixes allowed us to calculate AUTOCOD's performance metrics, like sensitivity.

**Results:**

AUTOCOD showed high sensitivity (≥0.75) in 10 chapters, with values above 0.90 for the three most common ones. The weighted-average of sensitivity showed no difference between periods without excess mortality and periods of excess mortality, a difference of 0.01 for periods of extreme mortality (+4 SD) and a difference of 0.04 for periods of extreme mortality (+6 SD). For the block classification, performance was similar.

**Conclusions:**

Even in periods of excess and extreme mortality, AUTOCOD accurately predicts the classification of the cause of death. Meaning that it is not affected by a potential loss in text-quality due to pressure in health services. This allows for the use of AUTOCOD for real time mortality surveillance and it highlights the importance of Artificial Intelligence as an advisory tool for Public Health policies in emergencies.

**Key messages:**

• Artificial Intelligence algorithms like AUTOCOD can predict the ICD-10 cause of death with very high sensitivity, during periods with and without excess mortality.

• Artificial Intelligence algorithms like AUTOCOD can be used for real-time cause specific mortality surveillance, providing valuable information for policy making during periods of excess mortality.

